# A novel arc geometry setting for pelvic radiotherapy with extensive nodal involvement

**DOI:** 10.1120/jacmp.v17i4.6028

**Published:** 2016-07-08

**Authors:** Maija Rossi, Eeva Boman, Tanja Skyttä, Mika Kapanen

**Affiliations:** ^1^ Medical Imaging Centre, Department of Medical Physics Tampere University Hospital Tampere Finland; ^2^ Department of Oncology Tampere University Hospital Tampere Finland

**Keywords:** anorectal carcinoma, vulvar cancer, treatment planning, arc therapy, wide fields

## Abstract

The aim of this study was to find optimal planning approach for large planning targets with complicated geometry requiring wide field openings. The study presents a novel approach for arc geometry design for pelvic targets with extensive nodal involvement. A total of 15 patients with anorectal carcinoma or vulvar cancer were selected retrospectively. For each patient, one seven‐field IMRT plan and three VMAT plans were calculated: one with two 360° arcs with no limitations for the field size (VMATw); one with two asymmetrically field‐size‐restricted 360° arcs (VMATr); and the proposed novel approach which consisted of one 360° arc with the field size restricted to the central PTV, and another arc divided into two 180° arcs, restricting the field sizes with the focus on the lymph nodes. The techniques were compared in terms of PTV coverage $\left(V_{\text{PTV}}(95\%)\right)$, dose maximum (D(max)), dose conformity index (CI), homogeneity index (HI), and organs at risk doses. The proposed novel approach with one full and two half arcs tended to have better PTV coverage ($V_{\text{PTV}}(95\%)=97\%\pm 2\%$, compared to 95%±3%,95%±3%, and 97%±2% in VMATw, VMATr, and 7f‐IMRT, respectively) and lower maxima (D(max)=107%±1%, compared to 110%±3%,110%±4%, and 110%±4% in VMATw, VMATr, and 7f‐IMRT, respectively); and lower or equal organs at risk doses. The superiority of the proposed technique (CI=1.16±0.05,HI=9±2) was more pronounced compared with the VMATw plans (CI=1.41±0.10, paired *t*‐test p<0.001;HI=12±2,p<0.001), but the proposed technique was slightly better also in comparison with the VMATr plans (CI=1.21±0.07,p<0.001;HI=11±4,p=0.015) and 7f‐IMRT plans (CI=1.18±0.03,p=0.016;HI=10±2,p=0.215). Radiotherapy treatment planning for large and complicated treatment volumes benefits not only from restricting the field size but also from careful field design that considers PTV geometry. This optimizes multileaf collimator movements, leading to better dose conformity and homogeneity.

PACS number(s): 87.53.Jw, 87.55.D, 87.56.jk:

## I. INTRODUCTION

Chemoradiation is widely used for the treatment of anorectal carcinoma and vulvar cancer instead of radical surgery, or postoperatively as adjuvant treatment in high‐risk patients with locally advanced disease.^1^, ^2^ The planning target volume (PTV) often encompasses the inguinal lymph nodes as well as the pelvic lymph node areas, making the shape of the PTV wide and complex. To achieve the required radiation dose conformity and homogeneity, it is essential to use detailed beam shaping. Techniques for delivering radiotherapy have evolved from three‐dimensional conformal radiotherapy (3D CRT) through intensity‐modulated radiation therapy (IMRT) to volumetric‐modulated arc therapy (VMAT). These developments offer the advantage not only in increased dose conformity, but also in improved sparing of organs at risk (OAR), including the small bowel, bladder, and rectum.^2^, ^3^, ^4^

In the current literature and clinical environment, VMAT is a widely used technique in radiotherapy at a variety of treatment sites.^(5)^ Several studies have shown that VMAT performs equally in PTV coverage, with equal or possibly improved OAR sparing compared with that obtained using 3D CRT and IMRT.^2^, ^6^, ^7^, ^8^, ^9^ In addition, VMAT delivers treatment with less beam‐on time and less monitor units (MUs) than IMRT. A typical field setting in a VMAT plan uses two 360° arcs. However, in the case of wide PTV this may result in nonoptimal multileaf collimator (MLC) movements in VMAT planning: first, due to hardware restrictions for MLC movements (i.e., the inability of two separate MLC openings simultaneously in the direction of leaf movements at a specific gantry angle (GA)); and second, due to the restricted MLC velocity from one GA to another. Furthermore, the physical limitations of the MLC geometry may reach extreme limits of MLC positions when attempting to cover distal parts of the PTV. In worst cases, this may also lead to MLC opening outside of PTV. The size of the PTV suggesting field opening considerably wider than 15 cm in the direction of the MLC movements, combined with the complicated geometry associated with the nodal involvement that may even leave holes in the beam's‐eye view at some angles, in many cases requires advanced VMAT planning.

Various approaches have been proposed to improve the dose distribution in large, complicated PTVs. First, limiting the field size in the direction of leaf movements to 15 cm has been proposed with good results.^2^, ^7^ This improves the VMAT plans but may still be suboptimal in comparison to an IMRT plan, especially if the PTV is large and has complicated geometry. Second, increasing the number of arcs is shown to improve the dose distribution.^5^, ^10^ However, in a busy clinical environment this might not be an optimal solution. Furthermore, the possibility for patient movement increases with the increasing treatment time. Another disadvantage is the increasing amount of small fields and the associated dosimetrical challenges. Third, for wide targets there is a possibility of using two isocenters. However, in the left–right axis this may lead to over‐ or underdose if there are any inaccuracies in the couch shifting.^(11)^ Couch shifting also increases the treatment time. Previous studies on VMAT use different numbers of arcs, or field size limitations. To the authors' knowledge there are no studies on a novel design of VMAT arcs that keep the number of arcs the same.

The aim of this paper is to introduce a method for VMAT optimization in large pelvic targets with extensive nodal involvement when a conventional two‐arc plan gives a suboptimal solution because of the size and complexity of the PTV. We propose a field design with one 360 arc, which has a field size limited to the proximal parts of the PTV, and another arc, divided into two partial 180° arcs, focused on the distal parts. The partial arcs include a collimator angle change at the GA of zero in order to adapt to the geometry of the PTV. Both the 360° arc and the partial 180° arcs are proposed to be restricted to 15 cm in the direction of leaf movements, or if dose coverage is not sufficient, to no more than 18 cm.

## II. METHODS

Fifteen consecutive patients with anal carcinoma, distal rectal carcinoma, or vulvar carcinoma with a need of bilateral inguinal and pelvic lymph node region radiotherapy were selected retrospectively for this study. The selected patients with anorectal carcinoma were originally described with two to three or four dose levels, depending on clinical characteristics; the irradiation of the PTV with large nodal involvement was followed by a booster to the primary tumor volume. The PTV was generated with a 7 mm margin to the CTV. The two first‐dose prescriptions for a given patient were technically challenging including the extensive lymph nodes, differing only in the superior PTV height. The booster to highest dose prescription was straightforward in planning. The patients with vulvar cancer had only one dose level with technically challenging PTV. The clinical doses prescribed for the patients varied, and these are presented in Table 1.

The patients were imaged with computed tomography in the treatment position. Imaging was performed with Philips Brilliance Big Bore (Philips Healthcare, Cleveland, OH) or Toshiba Aquilion LB (Toshiba America Medical Systems, Tustin, CA) using 3 mm slice thickness. Treatment planning was performed with the Eclipse treatment planning system (Aria 11, Varian Medical Systems, Palo Alto, CA) using coplanar treatment positions, and calculated with the analytical anisotropic algorithm (AAA 11.0.31) using 2.5 mm calculation grid. The treatment was planned for 6 MV photons of Varian Clinac iX linear accelerator (Varian Medical Systems) with Millennium 120 MLC (leaf width of 2.5–5 mm). Due to current experience and literature^2^, ^7^ with the VMAT plans, attention was kept on the field size of the x‐axis (FSx) which is the direction of the MLC movements.

**Figure 1:**
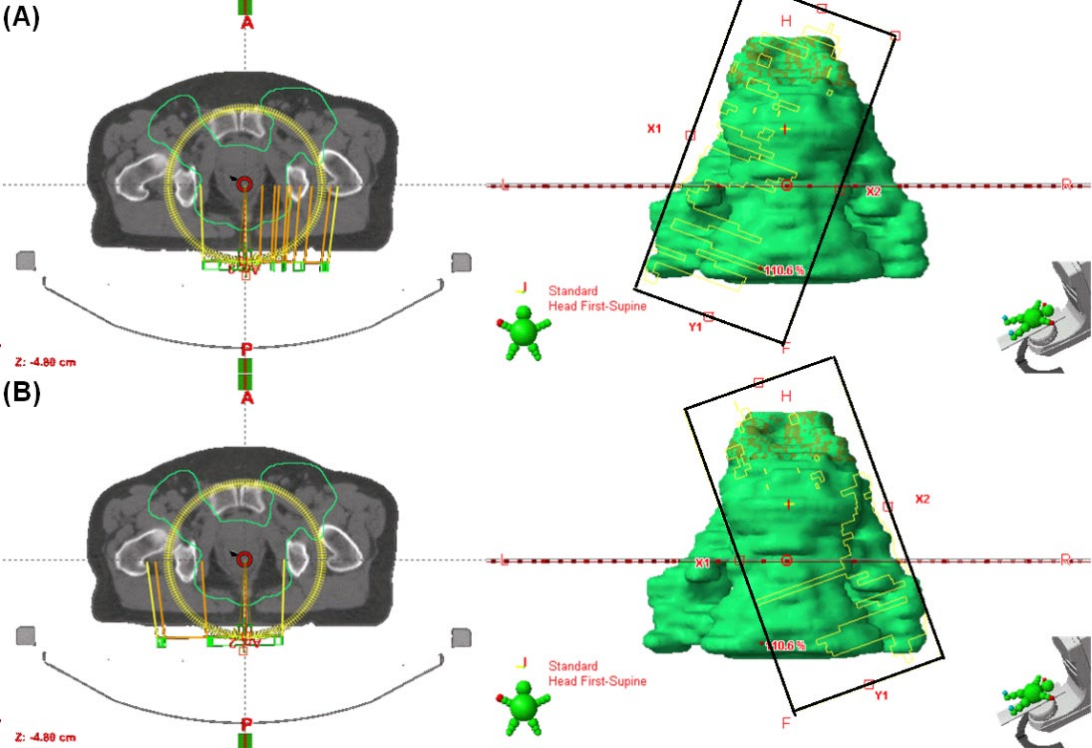
The geometry of the VMATr plans. The asymmetric design matches closely those in Fig. 2 (b) and (c), but with slightly less tilting of the collimator angles. Furthermore, if needed, both jaws are slightly closer to the isocenter in the VMATr than in the VMATr‐div planning to ensure proper coverage of the proximal PTV in VMATr. The FSx is limited to 15 cm or 18 cm.

**Figure 2:**
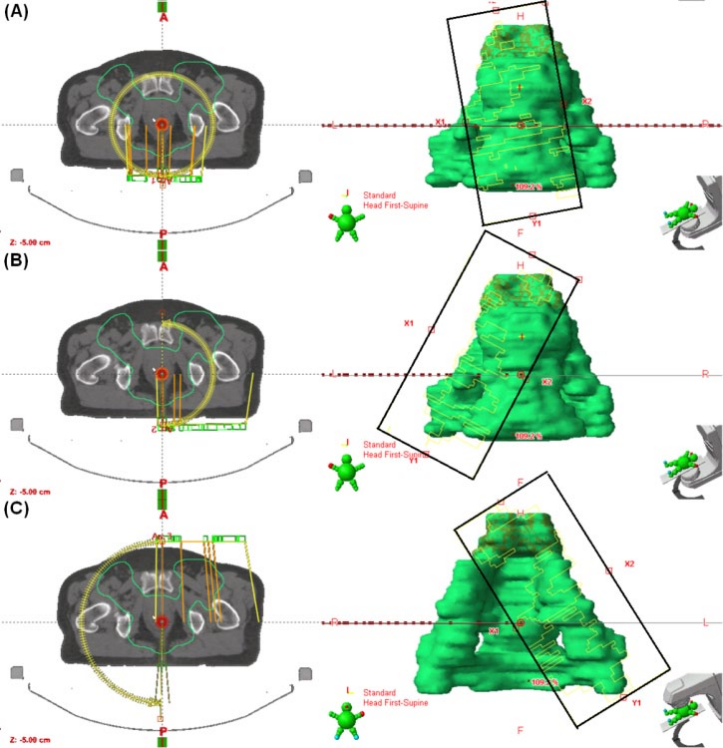
Geometry of the VMATr‐div plans. The first full arc (a) has subtle collimator angle tilting, and it treats mainly the proximal parts of the PTV. The second arc is divided into two halves (b and c), changing the collimator angle at the gantry angle of 0°. These are designed to treat mainly the distal lymph nodes, and collimator tilting is adjusted accordingly, ensuring proper lymph node coverage in all gantry angles. The FSx is limited to 15 cm or 18 cm.

Four different plans were created and compared. First, an IMRT plan with seven fields was planned (7f‐IMRT). The fields were directed at angles 204°, 256°, 308°, 0°, 52°, 104°, and 156°. The collimator angle was 0°. Each field was divided into two to four subfields due to the width of the PTV, with FSx varying according to PTV width given in Table 1. In the widest cases where three subfields were insufficient, two fields from the same angle were manually restricted to each side of the central axis, and these were each divided into two subfields by the software, resulting in four subfields from a given gantry angle. This is a problem with the treatment time and the main reason that we chose seven‐field IMRT instead of nine‐field IMRT. Although nine‐field IMRT might give better steepness of the DVH of the PTV, this may be compromised by patient motion caused by the increased treatment time. Second, a RapidArc plan with two wide full arcs was created (VMATw) as proposed by the Arc Geometry Tool in the planning system. Collimator angles of 10°–20° were used for the first arc as that resulted in smaller FSx than the 30° angle proposed by the Arc Geometry Tool; and the complement angle for the second arc. Third, a RapidArc plan with two arcs was created, but now the FSx was restricted to 15 cm (VMATr), as proposed in earlier studies.^2^, ^7^ Based on our experience, FSx of even 18 cm is still tolerable and allows for reasonable MLC movements. Therefore, in cases where the dose distribution resulting from the 15 cm restriction was unsatisfactory, 18 cm jaw opening was allowed. The jaw openings were slightly asymmetrically placed, as shown in Fig. 1. Fourth, a RapidArc plan with one full and two partial arcs was created (VMATr‐div), as shown in Fig. 2. The full‐rotation arc was targeted at the proximal part of the PTV, with the FSx restricted to 15 or 18 cm. The arc was designed with a moderate collimator angle to cover the proximal PTV at all angles, but different from zero to avoid tongue‐and‐groove effects. The two partial arcs were laterally asymmetrical, focusing on the distal lymph nodes, also with the FSx restricted to 15 or 18 cm, and the collimator angle adjusted to the PTV geometry as illustrated in Fig. 2 (b) and (c). In some cases, the collimator angle was closer to zero than in Fig. 2 (b) and (c), to ensure proper PTV coverage. For each patient case, optimal optimization criteria were searched for the PTV and OAR in the VMATr‐div plan by an experienced physicist. The optimization was then started from the beginning, keeping the criteria consistent in all the four plans, and the intermediate dose calculation was used.

**Table 1:** Patient characteristics

*Patient*	*Age (y)*	*Gender*	*Diagnosis*	*Total Doses of PTVs (Gy)*	*PTV Volume (cm^3^)*	*PTV Width (cm)*
A1	57	m	Anal carcinoma T2N3 stage 3B	39.6 / 45 / 50.4 / 59.4^a^	3086	28
A2	57	f	Anal carcinoma T2N1 stage 3A	36 / 45 / 54^a^	2268	24
A3	60	f	Anal carcinoma T2N1 stage 3A	30.6 / 45 / 54^a^	2221	23
A4	65	m	Anal carcinoma T4N3M1 stage 4	30.6 / 45 / 59.4^a^	2637	21
A5	74	f	Distal rectal adenocarcinoma T4N2M1 stage 4	45 / 50.4^a^	2421	22
A6	75	m	Anal carcinoma T2N0M0	36 / 45 / 54^a^	2528	28
A7	70	f	Distal rectal and anal adenocarcinoma T4N1 stage 3B	45 / 50.4^a^	2459	34
A8	46	f	Anal carcinoma T1N0 stage 1	45 / 50.4^a^	1868	28
A9	59	f	Anal carcinoma T3N0 stage 2	30.6 / 45 / 54^a^	2487	21
V1	78	f	Vulvar carcinoma T2N1a stage 3A	50.4^a^	1698	28
V2	83	f	Vulvar carcinoma T2N2c stage 3C	50^b^	1645	27
V3	68	f	Vulvar carcinoma T2N2b stage 3B	56^b^	2041	25
V4	69	f	Vulvar carcinoma T3N3 stage 4a	55c,d	5424	35
V5	69	f	Vulvar carcinoma T2N0 stage 2	50^b^	2238	23
V6	62	f	Vulvar carcinoma T1bN2b stage 3b	50.4^a^	3704	30

a ^a^ 1.8 Gy/fraction; ^b^ 2 Gy/fraction; ^c^ 2.2 Gy/fraction; ^d^ palliative.

In each patient, the volume $V_{\text{PTV}}$ and the maximum width of the PTV were measured. For each plan $V_{\text{PTV}}(95\%)$ and $V_{\text{BODY}}(95\%)$ were measured in cubic centimeters (cc). The PTV width was defined as the maximum width in the beam's eye view at GA of zero. Other parameters were measured in relative doses and volumes, including $D_{\text{PTV}}(98\%),D_{\text{PTV}}(2\%),V_{\text{PTV}}(95\%),V_{\text{CTV}}(95\%),V_{\text{PTV}}(105\%)$, and D(max), for the PTV and clinical target volume (CTV). The Paddick conformity index (CI) was used in the analysis using the equation $\text{CI}=(V_{\text{BODY}}{\left(95\%\right)}^{*}V_{\text{PTV}})/{\left(V_{\text{PTV}}(95\%)\right)}^{2}$.^(12)^ For CI, low values (CI≥1) indicate conformal dose distribution. Homogeneity index (HI) was calculated as in ICRU‐83, $\text{HI}=(D_{\text{PTV}}(98\%)-D_{\text{PTV}}(2\%))/{\left(D_{50}\right)}^{*}100$, where $D_{50}$ was the median dose to the PTV.^(13)^

For the OAR, the parameters $V_{\text{OAR}}90\%,V_{\text{OAR}}70\%V_{\text{OAR}}50\%$, and $V_{\text{OAR}}30\%$ were measured, where the OAR included the bladder, small bowel, and in the vulvar plans the rectum. The percentage OAR doses can be converted into Grays based on the first dose level which was the PTV used in the planning. The second (or third, in patient A1) dose level would be geometrically similar, except for the less superior PTV resulting in less dose in the small bowel. Because the total dose levels were different, the OAR doses were measured in percentages; that is to say, in a 50 Gy plan corresponding to physical doses of 45, 35, 25, and 15 Gy. Because most of the OAR constrains are designed for higher doses, in this study we aimed at “as low as reasonably achievable” with the OARs, and for easier comparisons between different prescriptions, percentage doses were measured.

Statistical analyses were computed with SPSS (v22.0.0.0). The normality of data distribution was tested with one‐sample Kolmogorov‐Smirnov test. Based on the normality, either parametric or nonparametric statistical analysis was computed. Comparisons between planning techniques were analyzed with paired *t*‐test, or, in case of nonnormal distribution, with Wilcoxon signed ranks test for all plans for $V_{\text{PTV}}(95\%),V_{\text{CTV}}(95\%),V_{\text{PTV}}(105\%),D(\text{max}),\text{CI},\text{HI},V_{\text{BLADDER}}(90\%),V_{\text{BLADDER}}(70\%),V_{\text{BLADDER}}(50\%),V_{\text{BLADDER}}(30\%);V_{\text{BOWEL}}(90\%),V_{\text{BOWEL}}(70\%),V_{\text{BOWEL}}(50\%)$, and $V_{\text{BOWEL}}(30\%)$; and for the vulvar plans additionally for $V_{\text{RECTUM}}(90\%),V_{\text{RECTUM}}(70\%),V_{\text{RECTUM}}(50\%)$, and $V_{\text{RECTUM}}(30\%)$. These variables were also tested for correlations with PTV volume and width with either Pearson correlation or Spearman's rho test, depending on the Kolmogorov‐Smirnov test‐given normality of the data: p<0.01 was considered significant, and p<0.05 was considered as a tendency.

## III. RESULTS

The patient characteristics are presented in Table 1. For each patient, all four alternative plans were created. However, in several cases the dose distributions of the VMATw were highly inappropriate for treatment, especially for the vulvar patients. Three vulvar patient cases could not be designed with the 15 cm jaw opening due to poor PTV coverage. In these cases, both the VMATr and the VMATr‐div plans were successfully planned with 18 cm jaw opening for the analyses. The number of monitor units were highest (p<0.001) in the IMRT plans (1980±500); similar (p=0.387) in the VMATr (700±110) and VMATr‐div (680±90); and lowest (p<0.001) in the VMATw (350±50) plans. The MU reflects the amount of modulation.

The time to generate the VMAT plans was approximately the same. The design of dividing the second arc took only a few minutes longer in the VMATr‐div planning than in the VMATw and VMATr plans where this division was not designed. The IMRT planning required similar time in designing the fields in the easiest cases. The wide PTVs that required manually dividing fields for a given gantry angle required more time, especially because in several cases the error was detected only after the optimization. IMRT optimization was faster than VMAT optimization. However, after the optimization VMATr‐div plans were the most ready for treatment after the first optimization; others would require more planning for enhancing the dose distribution, meaning both treating dose minima inside the PTV and removing dose maxima.

### A. PTV coverage

**Figure 3:**
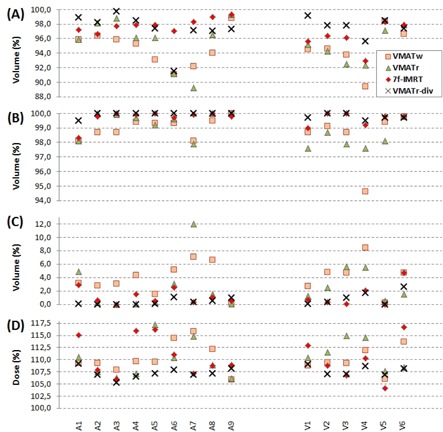
Descriptive figures of the relative dose coverage from the dose‐volume histogram are shown for the anorectal (A1–A9) and vulvar (V1–V6) plans. The low doses in the target volumes are presented as $V_{\text{PTV}}(95\%)$ and $V_{\text{CTV}}(95\%)$ in (a) and (b), respectively. The doses representing the maxima are given as $V_{\text{CTV}}(105\%)$ and D(max%) in (c) and (d), respectively.

Dose statistics of the PTV and CTV are shown in Fig. 3. Analyzing the 15 patient cases, $V_{\text{PTV}}(95\%)$ was the highest in the VMATr in one patient (similar to 7f‐IMRT in patient A9); in the 7f‐IMRT plan in six patients; and in VMATr‐div in nine patients. Within the measurement accuracy of 0.1%, there were several plans having $V_{\text{CTV}}(95\%)$ close to 100%, and therefore, in four patients two plans, and in three patients three plans were equally good. This resulted in the best CTV coverage in the VMATw in two patients; in the VMATr in three patients; in the 7f‐IMRT plan in seven patients; and in the VMATr‐div in thirteen patients. At the other end of the DVH, $V_{\text{CTV}}(105\%)$ was the lowest in the VMATr in two patients; in the 7f‐IMRT plan in five patients; and in the VMATr‐div in twelve patients with four ties at the accuracy of the measurement of 0.1%. D(max) was the lowest in the VMATw plan in two patients; in the 7f‐IMRT plan in two patients; and in the VMATr‐div in eleven patients. The superiority of $V_{\text{CTV}}(105\%)$ and D(max) was not always associated to the same planning technique in a given patient due to variations in the shape of the DVH at high doses.

Statistical test of the described preferences of technique in the anorectal cases are shown in Table 2. In the nine anorectal plans, higher D(max) in the 7f‐IMRT than in the VMATr‐div plans reached statistical significance. Superiority of VMATr‐div over 7f‐IMRT and VMATr in CTV coverage was also shown although the differences were small. The CI of VMATr was lower than those of 7f‐IMRT and VMATr‐div. The VMATw plans were clearly of lower quality than any of the three other plans (Fig. 3), and they were excluded from the statistical analyses in both the anorectal and vulvar cases.

**Figure 4:**
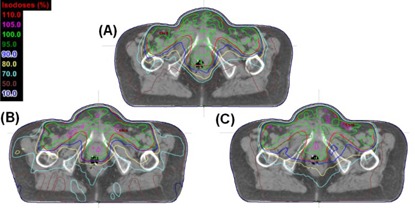
Example of the comparison between VMATr‐div (a) 7f‐IMRT (b), and VMATr (c) plans in a vulvar cancer treatment (patient V4) with large nodal involvement in the PTV, contoured red in the figures. The VMATr‐div plan has homogeneous dose to the PTV, associated with smaller volumes receiving lower doses outside the PTV (e.g., 50% (brown) and 70% (light blue)).

**Figure 5:**
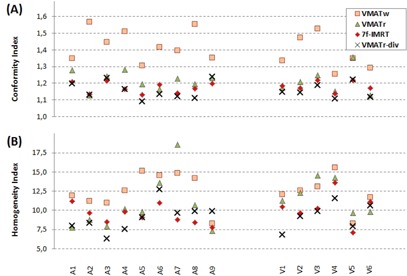
Conformity index (a) and homogeneity index (b) are shown for the anorectal (A1–A9) and vulvar (V1–V6) plans for each patient.

An example of one patient with vulvar cancer is shown in Fig. 4, presenting the 7f‐IMRT, VMATr, and VMATr‐div techniques. The associated DVH statistics of the six vulvar plans are detailed in Table 3. The VMATr‐div plan was generally the best and VMATr plan the worst, with the 7f‐IMRT in between (Fig. 3). This was shown by the statistical tests disfavoring VMATr in $V_{\text{PTV}}(95\%)$ and $V_{\text{CTV}}(95\%)$ (Table 3). Also higher D(max) in VMATr than in VMATr‐div was supported statistically. Only in one patient (V6) the PTV of the VMATr‐div seemed slightly lower at the V(95%) (Fig. 3), but also this patient had the best CI in the VMATr‐div plan (Fig. 5(a)).

**Table 2:** Comparisons between the planning techniques in the anorectal carcinoma using the Student's *t*‐test for paired samples

	*7f‐IMRT* (mean±SD)	*VMATr* (mean±SD)	*VMATr‐div* (mean±SD)	*7f‐IMRT ‐ VMATr (p)*	*7f‐IMRT ‐ VMATr‐div (p)*	*VMATr ‐ VMATr‐div (p)*
$V_{\text{PTV}}(95\%)$ (%)	98±1	96±3	97±2	0.050^a^	0.593^a^	0.051^a^
$V_{\text{PTV}}(95\%)$ (%)	100±1	99±1	100±0	0.202^a^	0.027^a^,^b^	0.028^a^,^b^
$V_{\text{CTV}}(105\%)$ (%)	1.1±1.0	2.5±4.0	0.3±0.4	0.333	0.054	0.146
D(max) (%)	111±4	110±4	107±1	0.555	0.018^b^	0.070
CI	1.17±0.03	1.22±0.05	1.16±0.05	0.029^b^	0.247	0.008^b^
HI	9±1	11±4	9±2	0.379	0.653	0.199
$V_{\text{BLADDER}}(90\%)$	26±14	31±18	28±14	0.019^b^	0.029^b^	0.158
$V_{\text{BLADDER}}(70\%)$	60±14	68±20	66±17	0.037^b^	0.050	0.678
$V_{\text{BLADDER}}(50\%)$	87±15	90±15	95±8	0.730	0.008^a^,^b^	0.249^a^
$V_{\text{BLADDER}}(30\%)$	99±3	96±12	100±0	0.715^a^	0.109^a^	0.109^a^
$V_{\text{BLADDER}}(90\%)$	9±11	8±10	9±11	0.672^a^	1.000^a^	0.753^a^
$V_{\text{BLADDER}}(70\%)$	24±17	22±18	24±19	0.368	0.844	0.169
$V_{\text{BLADDER}}(50\%)$	55±24	63±32	61±29	0.273	0.124	0.705
$V_{\text{BLADDER}}(30\%)$	79±28	83±29	79±29	0.404	0.775	0.726^a^

a ^a^ Data distribution not normal, Wilcoxon signed‐ranks p‐value is given.

b ^b^ p<0.05.

**Table 3:** Comparisons between the planning techniques in the vulvar cancer using the Student's *t*‐test for paired samples

	*7f‐IMRT* (mean±SD)	*VMATr* (mean±SD)	*VMATr‐div* (mean±SD)	*7f‐IMRT ‐ VMATr (p)*	*7f‐IMRT ‐ VMATr‐div (p)*	*VMATr ‐ VMATr‐div (p)*
$V_{\text{PTV}}(95\%)$ (%)	96±2	95±2	98±1	0.061	0.057	0.020^b^
$V_{\text{CTV}}(95\%)$ (%)	100±0	98±1	100±0	0.006^b^	0.346	0.007^b^
$V_{\text{CTV}}(105\%)$ (%)	1.3±1.8	2.8±2.2	1.0±1.0	0.276	0.407	0.086
D(max) (%)	110±4	111±3	108±1	0.607	0.232	0.042
CI	1.18±0.03	1.21±0.08	1.15±0.04	0.348	0.007^b^	0.031^b^
HI	10±2	12±2	9±2	0.102	0.163	0.023^b^
$V_{\text{BLADDER}}(90\%)$	34±40	32±38	37±39	0.500^a^	0.251	0.345^a^
$V_{\text{BLADDER}}(70\%)$	63±29	70±27	71±27	0.155	0.124	0.763
$V_{\text{BLADDER}}(50\%)$	92±8	92±9	87±11	0.959	0.206	0.169
$V_{\text{BLADDER}}(30\%)$	99±1	100±1	99±2	0.317^a^	0.317^a^	0.317^a^
$V_{\text{BOWEL}}(90\%)$	10±12	8±8	10±14	0.276	0.904	0.403
$V_{\text{BOWEL}}(70\%)$	28±26	25±25	26±26	0.008^b^	0.080^b^	0.208
$V_{\text{BOWEL}}(50\%)$	51±32	47±31	44±29	0.028^a^,^b^	0.028^a^,^b^	0.075^a^
$V_{\text{BOWEL}}(30\%)$	69±30	68±29	68±29	0.445	0.193	0.928
$V_{\text{RECTUM}}(90\%)$	29±36	31±35	32±34	0.136^a^	0.080^a^	0.080^a^
$V_{\text{RECTUM}}(70\%)$	47±27	51±26	48±27	0.225^a^	0.345^a^	0.286
$V_{\text{RECTUM}}(50\%)$	76±16	68±23	66±22	0.192	0.134	0.423
$V_{\text{RECTUM}}(30\%)$	100±1	98±3	97±5	0.285^a^	0.109^a^	0.593^a^

a ^a^ Data distribution not normal, Wilcoxon signed‐ranks p‐value is given.

b ^b^ p<0.05.

The conformity index is shown in Fig. 5(a). Besides the test statistics shown in Table 2 for the anorectal and vulvar cases separately, statistics on the whole group of 15 patients are described below. The CI was the lowest, indicating the best conformity, in the VMATr plan in patient A2 (similar to VMATr‐div); in the 7f‐IMRT in four patients (similar to VMATr‐div in patient V5); and in the VMATr‐div plan in twelve patients. The VMATr‐div plan was better than the VMATr plan in 13 patients (p<0.001), and better than the 7f‐IMRT plan in 11 patients (p=0.016). The 7f‐IMRT plan was better than the VMATr plan in 11 patients (p=0.019). The CI was worst in every patient for VMATw plan (p<0.000), except for being closely similar to VMATr in patient V5.

The HI (Fig. 5(b)) for the whole group of 15 patients was the lowest, indicating the best homogeneity, in the VMATr plan in three patients; in the 7f‐IMRT in four patients (similar to VMATr‐div in patient V5); and in the VMATr‐div plan in ten patients. The HI was equally good in the VMATr‐div and 7f‐IMRT plans (p=0.215). The VMATr‐div was slightly better than the VMATr plan (p=0.015), but the difference between VMATr and 7f‐IMRT was not significant (p=0.112). VMATw was worse than the three other plans in terms of HI (p<0.000), except for the smaller difference (p=0.039) between VMATw and VMATr.

### B. Organs at risk

**Figure 6:**
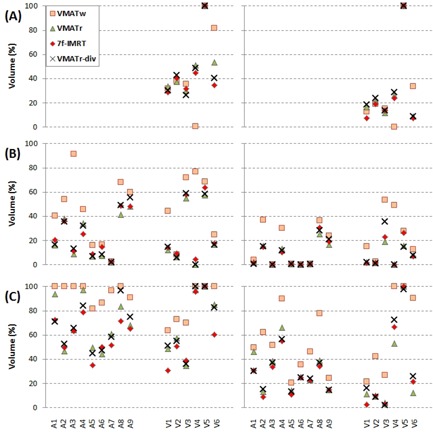
Doses for V(70%) and V(90%) are shown at left and right, respectively, for rectum (a), small bowel (b), and bladder (c). The statistics are shown individually for the anorectal carcinoma patients (A1–A9) and vulvar patients (V1–V6).

The DVH characteristics for the higher doses (70% and 90%) of the OARs are presented in Fig. 6, and the associated statistics of all the recorded dose volumes in Tables 2 and 3 for the anorectal and vulvar cases, respectively. The organs at risk tended to receive the highest doses in the plans generated with the VMATw protocol (Fig. 6), and they were dismissed from the tables. The 7f‐IMRT, VMATr, and VMATr‐div plans were in most cases not significantly different from each other. In the anorectal carcinoma cases, the 7f‐IMRT technique seemed slightly better than the VMAT techniques in the bladder (Table 2), but the difference was minimal (Fig. 6). In the vulvar cases, the $V_{\text{BOWEL}}(70\%)$ and $V_{\text{BOWEL}}(50\%)$ was slightly better in both VMAT plans than in the 7f‐IMRT plans. Although not statistically shown, in several cases of the vulvar plans the OARs received higher doses in the VMATr‐div than in the VMATr plans (Fig. 6). In the complex geometries of the vulvar plans, the VMATr technique was unable to cover the PTV properly, leading to lower doses in the OAR's but associated with underdose in the PTV. An example of this in the rectum is shown in Fig. 4.

### C. Correlations

In the anorectal carcinoma patients, Pearson correlation was found between $V_{\text{CTV}}(105\%)$ and the PTV width (Pearson r=0.903,p=0.001) in the VMATr. The PTV width also tended to correlate negatively with $V_{\text{CTV}}(95\%)$ in the VMATr plans, but only in the Pearson correlation test (r=−0.678,p=0.045). In $V_{\text{CTV}}(95\%)$ there was a tendency for negative correlation with PTV volume in the 7f‐IMRT plan (Spearman's rho r=−0.735,p=0.024). Positive tendency between PTV width and HI was found in the VMATr plans (r=0.716,p=0.013).

**Figure 7:**
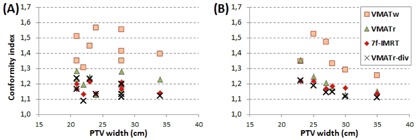
Correlation between conformity index and PTV width in the anorectal carcinoma (a) and vulvar cancer (b) patients.

In the vulvar plans, the PTV volume tended to correlate negatively with $V_{\text{PTV}}(95\%)$ (r=−0.894,p=0.016) in the Pearson correlation test in the VMATr‐div plans. The CI tended to correlate negatively with the PTV width in each the 7f‐IMRT plans (r=−0.924,p=0.008), the VMATr plans (r=−0.818,p=0.047), and the VMATr‐div plans (r=−0.901,p=0.014). Positive correlation between PTV width and HI was found only in the IMRT plans (r=0.939,p=0.006). The CI correlation is shown in Fig. 7.

## IV. DISCUSSION

Large PTV's with complicated geometry including nodal involvement are problematic in clinical treatment planning. In IMRT, cold‐ and hot‐spots are usually seen, the number of monitor units is large, and the fields are divided into two to even four carriage groups. Beside inhomogeneous dose distribution, this means long treatment times. The issue with long treatment times has been managed with the advent of VMAT techniques, and the dose distribution has remained equal or improved when the field size has been limited to 15 cm.^2^, ^7^ Further improvements in the dose distribution may be achieved with the addition of further arcs,^5^, ^10^ but in terms of in‐room time this is a drawback. Another issue with the increasing number of arcs is the increasing amount of leakage radiation as the amount of modulation increases and smaller MLC openings are used. Asymmetrical, FSx‐restricted techniques, such as VMATr in the present study, may provide good dose coverage and conformity for PTVs considerably wider than 15 cm, but when associated with the complicated geometry of the nodal involvement, we suggest improvements with a novel geometrical arc setting.

This paper suggests an arc technique with one full and two half arcs, which has three important advantages. First, no increment in the amount of scattered radiation, in terms of increased number of treatment angles, is introduced. Although the number of arcs is increased to three, the number of arc degrees used for planning remains equal to two full arcs (i.e., 720°). Second, the dose distribution in both the PTV and OARs is better, or at least equal, in all cases. Third, with the first half arc starting from the GA of zero, followed by the full arc, and finally coming back to zero with the second half arc, rapid treatment may be completed with efficient use of machine time and reduced probability for intrafractional patient movement. If the patient is imaged prior to treatment, this benefit is partly lost as the gantry is usually not at the GA of zero after imaging; however, the last arc still ends with the gantry conveniently at zero. In the sense that the proposed technique used asymmetrical fields in order to consider the PTV geometry, it is slightly reminiscent of one described earlier with breast treatments, with two 190° arcs designed to treat at different geometrical locations.^14^, ^15^ Due to different PTV geometry, the present study, however, had more beam angles and the PTV coverage was ensured not only by restricting the FSx to both sides of the central axis, but also by adjusting the collimator angles.

Whereas comparisons between IMRT and VMAT have different views as to the superiority of one over another,^(5)^ earlier studies have unanimously shown that wide arcs that cover the entire PTV at all gantry angles may not be the best solution in cases where the resulting jaw opening exceeds the 15 cm limit.^2^, ^7^ This study agrees with both of these findings. First, the comparisons between IMRT and VMAT plans were not consistent, favoring IMRT in some patients and VMAT in others. The difference in many patients was slight and preference depended on whether CI, HI, or other parameters were analyzed. Second, the VMAT plan with two full arcs was clearly better with restricted, than unrestricted, jaw opening. Thus, considering only techniques presented in previous literature, provided that the jaw opening was restricted, the VMAT performed well but not better in comparison to IMRT.

Besides confirming previous literature we introduced a novel technique closely similar to the two‐arc plan with the FSx restricted at 15 or 18 cm, but better taking into account the PTV geometry. This was accomplished with the first arc treating mainly the proximal PTV, and the second treating the distal PTV, with the collimator angle changing at the GA of zero. In the three most challenging plans (i.e., 50% of the vulvar cases) it was also seen that increasing the jaw opening to 18 cm enabled sufficient PTV coverage where FSx of 15 cm resulted in dose minima inside the PTV; but unrestricted jaws did not permit conformal plan, either. The method presented works not only for anorectal cases, but for most or all different pelvis cases with extensive nodal involvement.

The organs at risk did not have a clear preference between planning techniques, apart from being clearly worst in the VMATw plans. IMRT is known for steep gradients in the favor of OARs around the PTV, which was true in some cases, especially for the higher doses (50%–90%) of the bladder in the anorectal but not the vulvar plans. However, with the mid doses (50%–70%) of the small bowel of the vulvar plans VMAT performed better. With the large PTVs requiring the dose to spread irrespective of the technique, no consistent statistical preference of technique was seen. It is possible that, if the optimization criteria had been changed between the plans (e.g., by using different optimization weights in the IMRT than the VMAT plans), the result might have been different. Such approach would have made comparisons between techniques biased, depending on the experience and skills of the planner. The results might have been compromised and changing the optimization criteria was therefore abandoned.

The major motive of this study was the challenge in planning large and complicated PTVs. These often lead to decreased dose conformity. Generally, correlations between the width and volume of the PTV to the DVH figures were few, with a mild trend towards lower dose coverage at 95% and higher maxima when the size of the target increased. However, the negative correlation between CI and PTV width in the vulvar cancer patients could not be expected. This was not seen in the anorectal carcinoma patient group, nor in the correlation with the PTV volume and, therefore, it is likely to derive from the patient cohort. Besides the size, the complexity of the PTV and the proximity of OARs are major factors in dose conformity.

This study is limited in the number of patients. However, both the anorectal and vulvar plans showed similar trend in favor of the novel approach. With the limited number of the investigated patients having large lymph node involvement, trends shown in the figures should be emphasized more than the p‐values, and these imply that the suggested technique is promising. The method has been implemented in our clinical practice for pelvic patients with extensive lymph node involvement.

## V. CONCLUSION

We propose a technique that retains the irradiation angles of two arcs, and designs the collimator angles individually to the patient anatomy. The arc geometry considers the proximal and distal parts separately, and the collimator angle of the second arc adjusts to the changing position of the distal parts at the gantry angle of zero. Benefits are thus achieved in the PTV dose conformity, the sparing of healthy tissue, and treatment time efficacy.

## ACKNOWLEDGMENTS

This study was supported by the Instrumentarium Foundation.

## COPYRIGHT

This work is licensed under a Creative Commons Attribution 3.0 Unported License.

## Supporting information

Supplementary Material

Supplementary Material

Supplementary Material

Supplementary Material

Supplementary Material

Supplementary Material

Supplementary Material

Supplementary Material

Supplementary Material

Supplementary Material

Supplementary Material

Supplementary Material

Supplementary Material

Supplementary Material
