# Supplementary material for: A novel arc geometry setting for pelvic radiotherapy with extensive nodal involvement
Source: J Appl Clin Med Phys. 2016 Jul 8;17(4):73–85. doi: 10.1120/jacmp.v17i4.6028 (PMC5690051; doi:10.1120/jacmp.v17i4.6028)
Supplement: Supplementary file 1 — Supplementary Material [file ACM2-17-073-s001.doc]

**A novel arc geometry setting for pelvic radiotherapy with extensive nodal involvement**

**Maija Rossi, Eeva Boman, Tanja Skyttä, Mika Kapanen**

Maija Rossi, PhD, Eeva Boman, PhD, Mika Kapanen, PhD

*Medical Imaging Centre, Department of Physics*

*Tampere University Hospital*

*Teiskontie 35, 33520 Tampere*

*Finland*

[maija.rossi@pshp.fi](mailto:maija.rossi@pshp.fi), [eeva.boman@pshp.fi](mailto:eeva.boman@pshp.fi), [mika.kapanen@pshp.fi](mailto:mika.kapanen@pshp.fi)

Maija Rossi, PhD, Eeva Boman, PhD, Tanja Skyttä, MD, Mika Kapanen, PhD

*Department of Oncology*

*Tampere University Hospital*

*Teiskontie 35, 33520 Tampere*

*Finland*

[tanja.skytta@pshp.fi](mailto:tanja.skytta@pshp.fi)

Arc geometry in large PTVs
