# Supplementary figures and images for: A novel arc geometry setting for pelvic radiotherapy with extensive nodal involvement
Source: J Appl Clin Med Phys. 2016 Jul 8;17(4):73–85. doi: 10.1120/jacmp.v17i4.6028 (PMC5690051; doi:10.1120/jacmp.v17i4.6028)

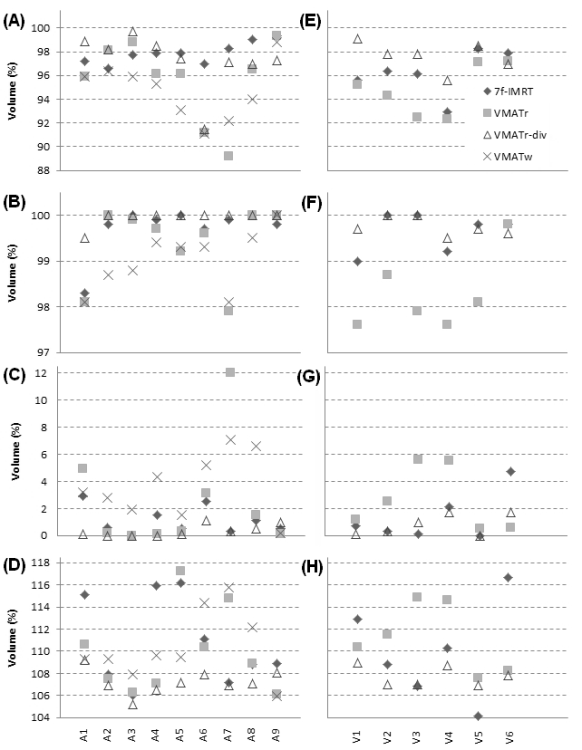

Supplement: Supplementary file 2 — Supplementary Material [file ACM2-17-073-s002.png]

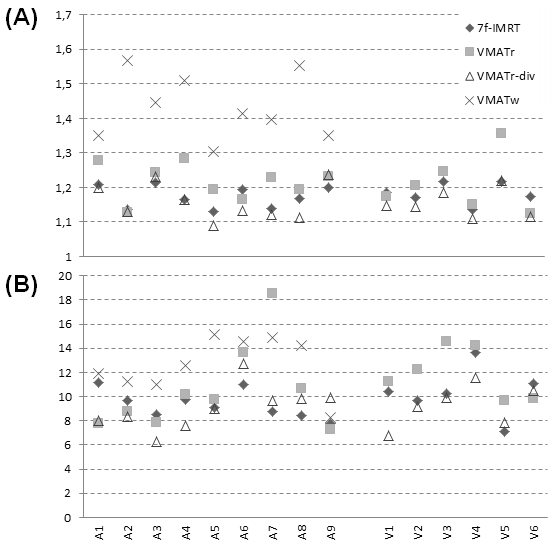

Supplement: Supplementary file 3 — Supplementary Material [file ACM2-17-073-s003.png]

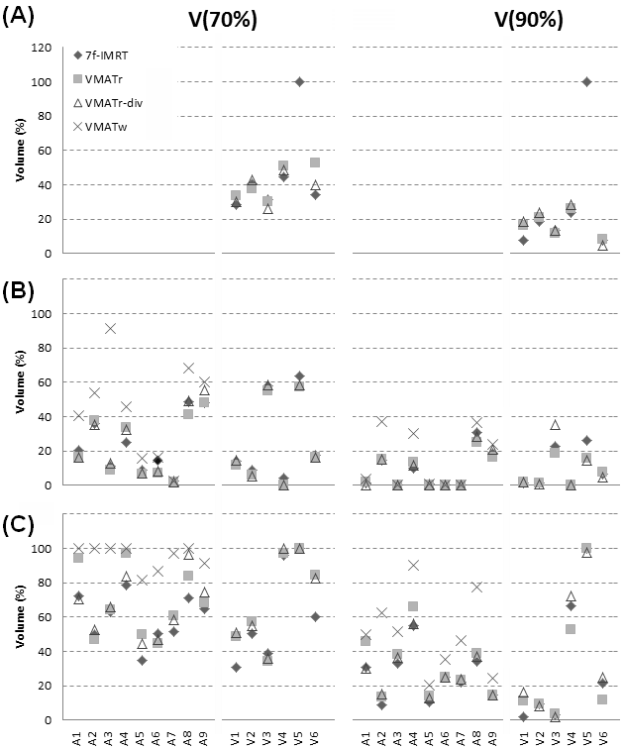

Supplement: Supplementary file 4 — Supplementary Material [file ACM2-17-073-s004.png]

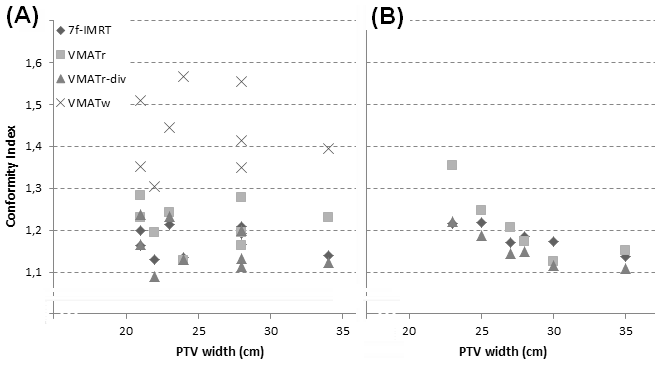

Supplement: Supplementary file 5 — Supplementary Material [file ACM2-17-073-s005.png]

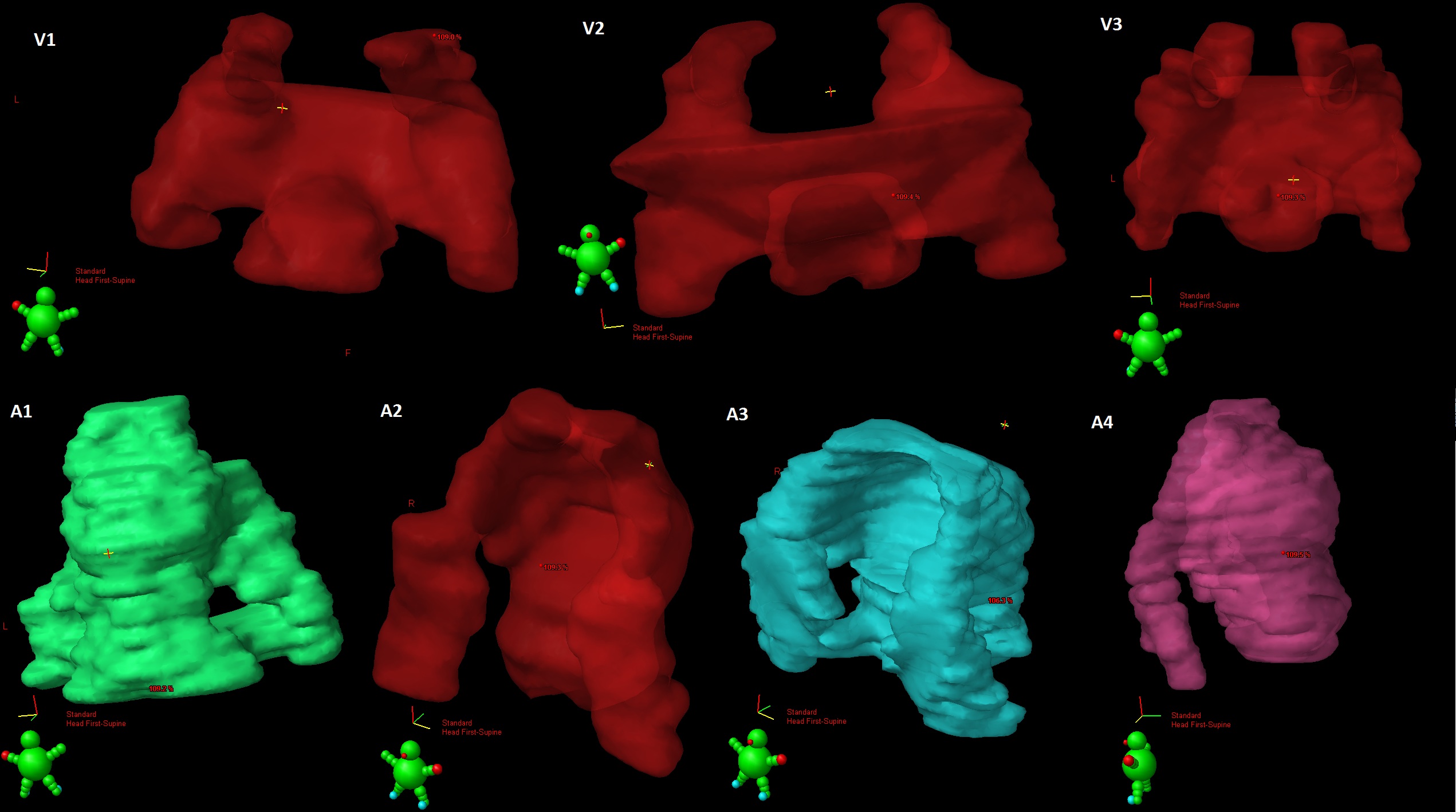

Supplement: Supplementary file 6 — Supplementary Material [file ACM2-17-073-s006.jpg]

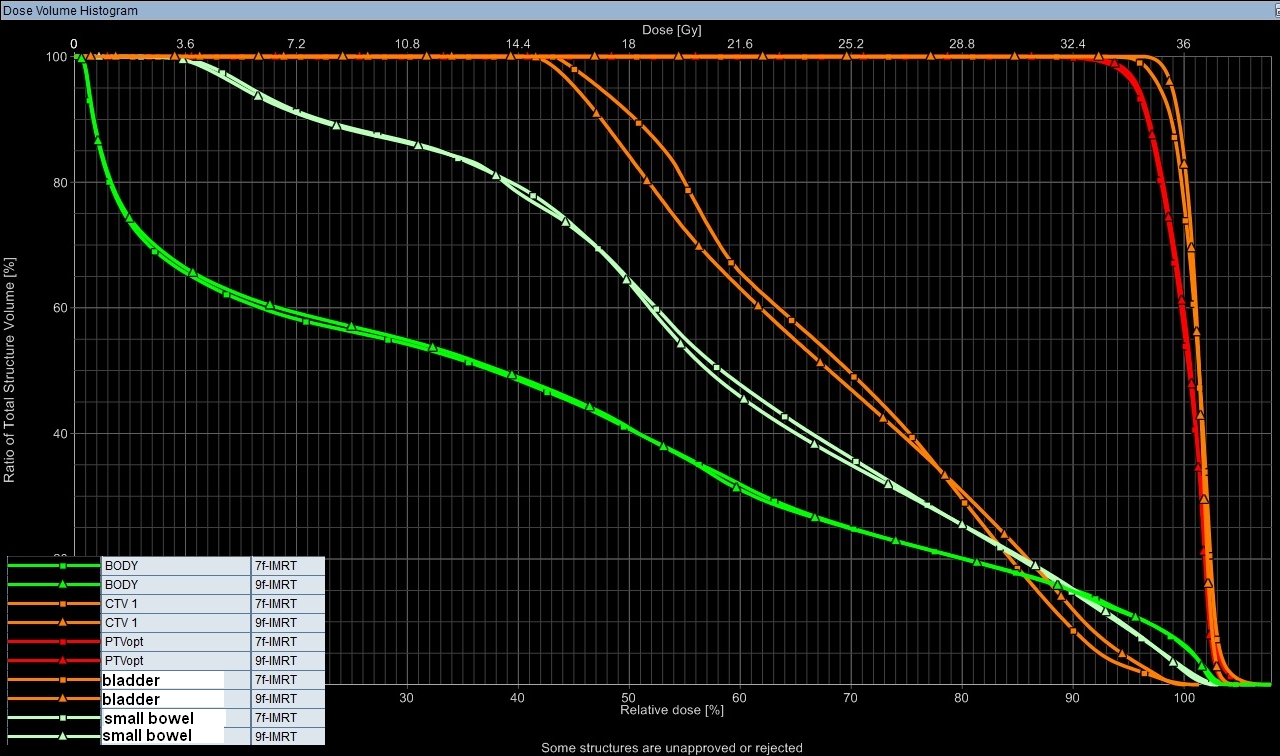

Supplement: Supplementary file 7 — Supplementary Material [file ACM2-17-073-s007.jpg]

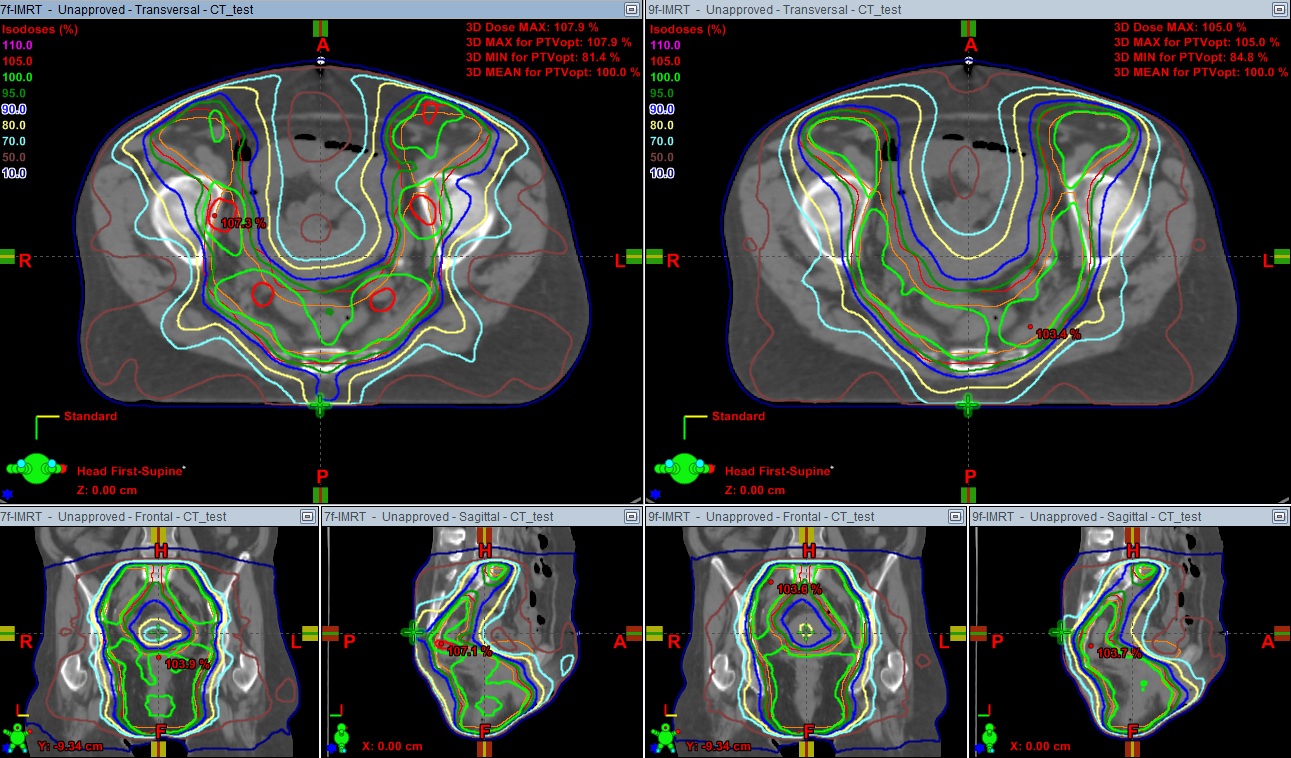

Supplement: Supplementary file 8 — Supplementary Material [file ACM2-17-073-s008.jpg]

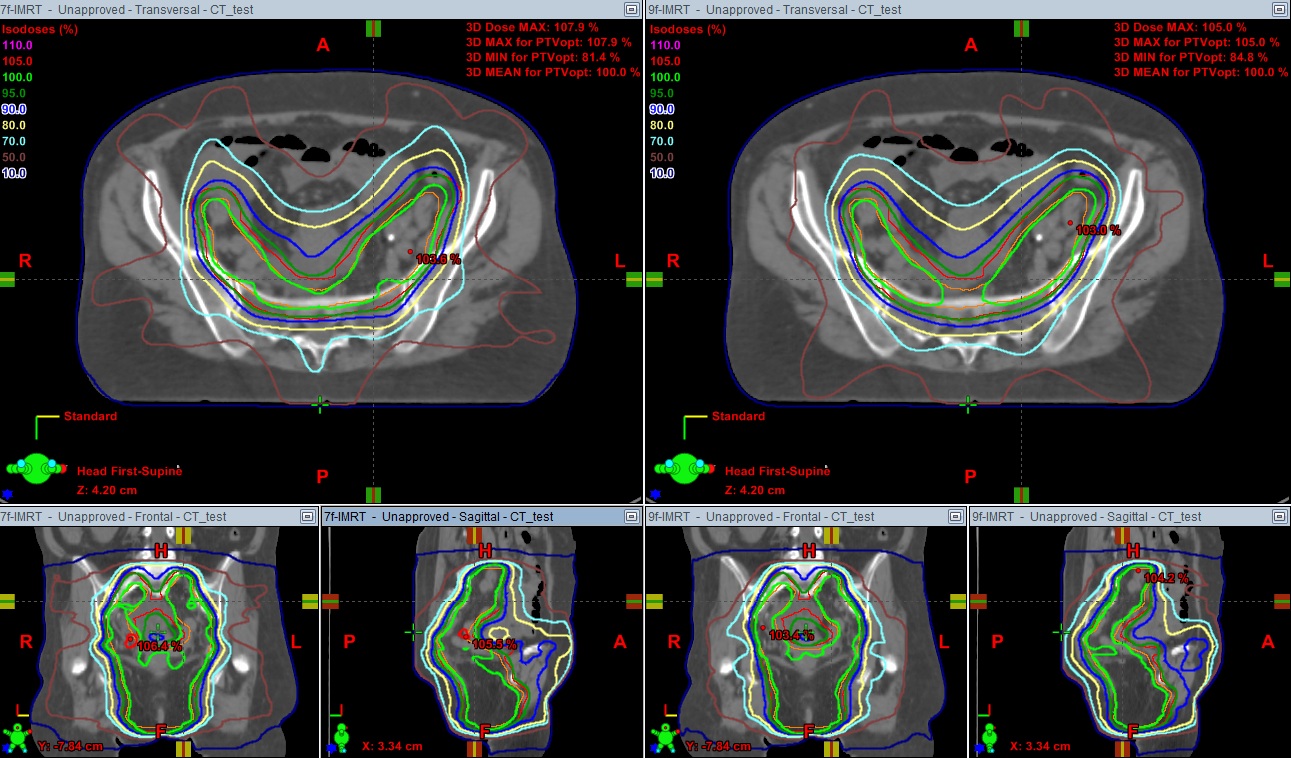

Supplement: Supplementary file 9 — Supplementary Material [file ACM2-17-073-s009.jpg]

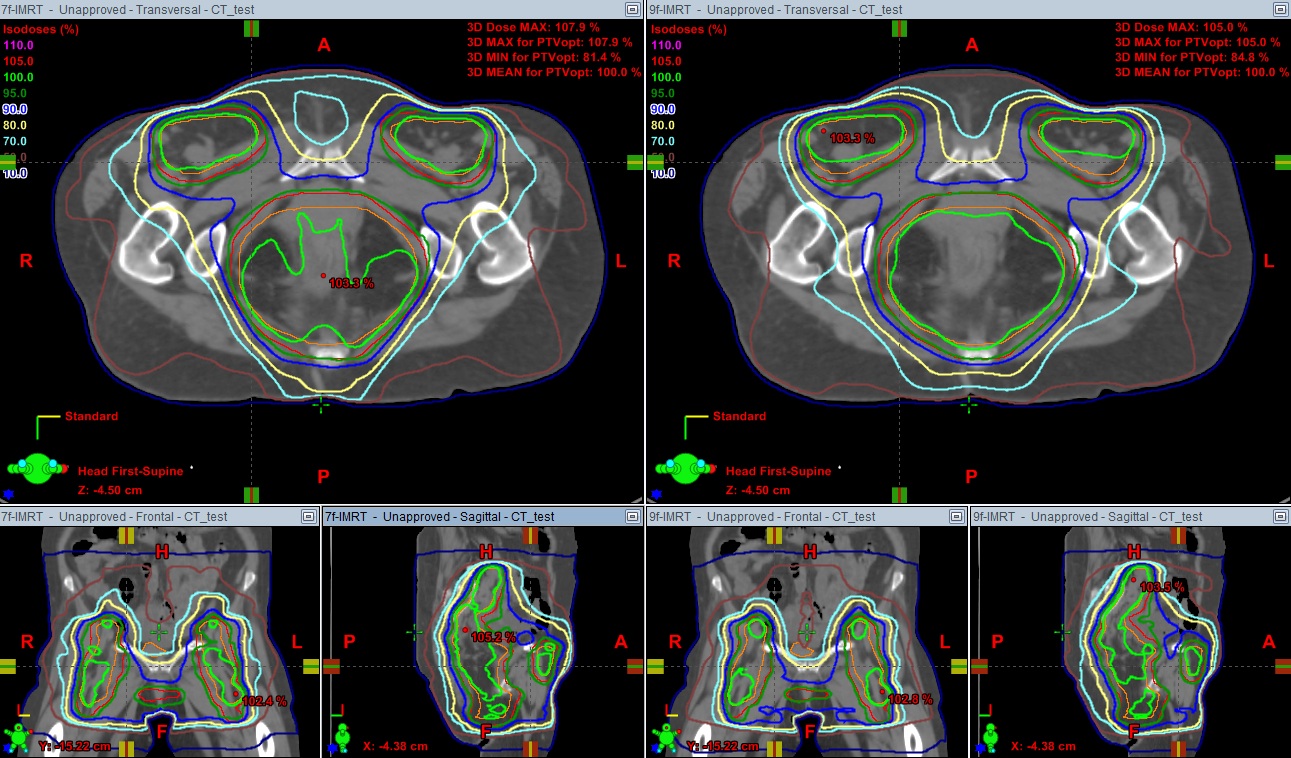

Supplement: Supplementary file 10 — Supplementary Material [file ACM2-17-073-s010.jpg]

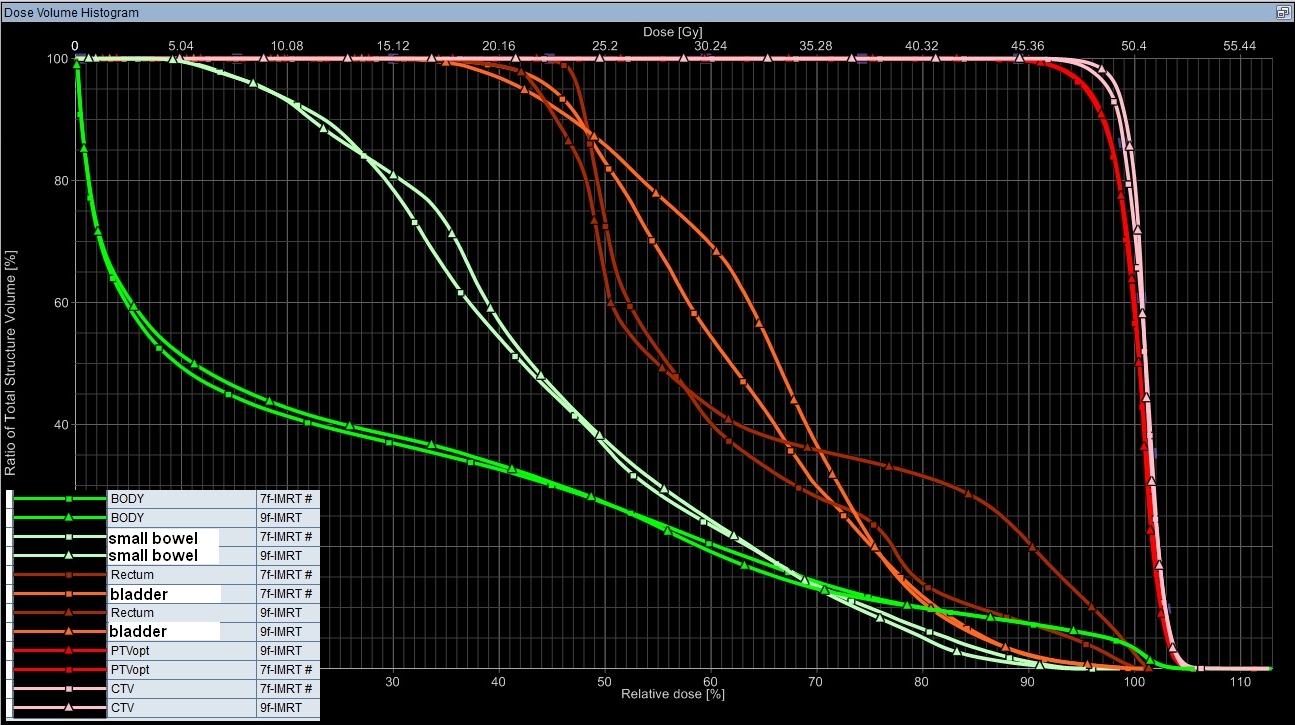

Supplement: Supplementary file 11 — Supplementary Material [file ACM2-17-073-s011.jpg]

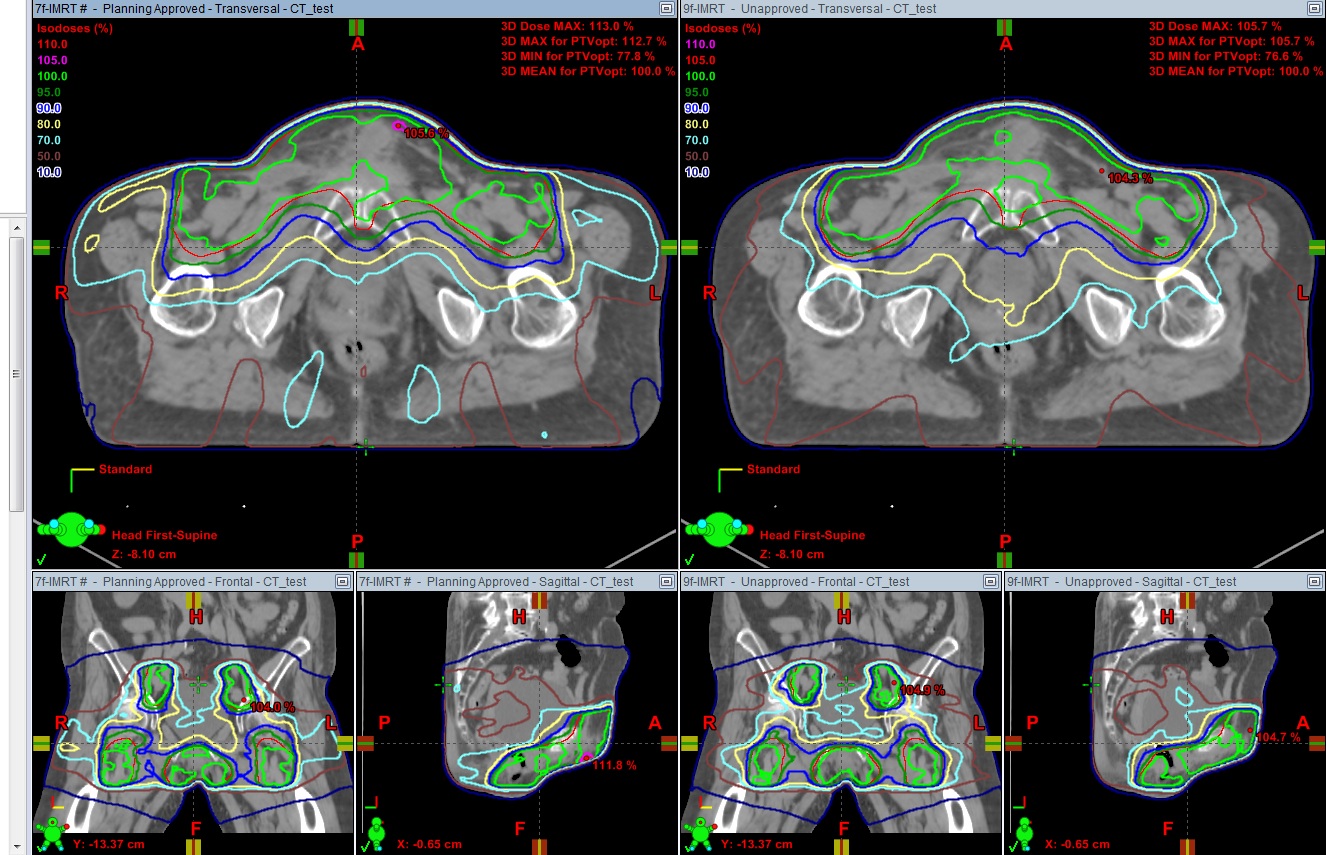

Supplement: Supplementary file 12 — Supplementary Material [file ACM2-17-073-s012.jpg]

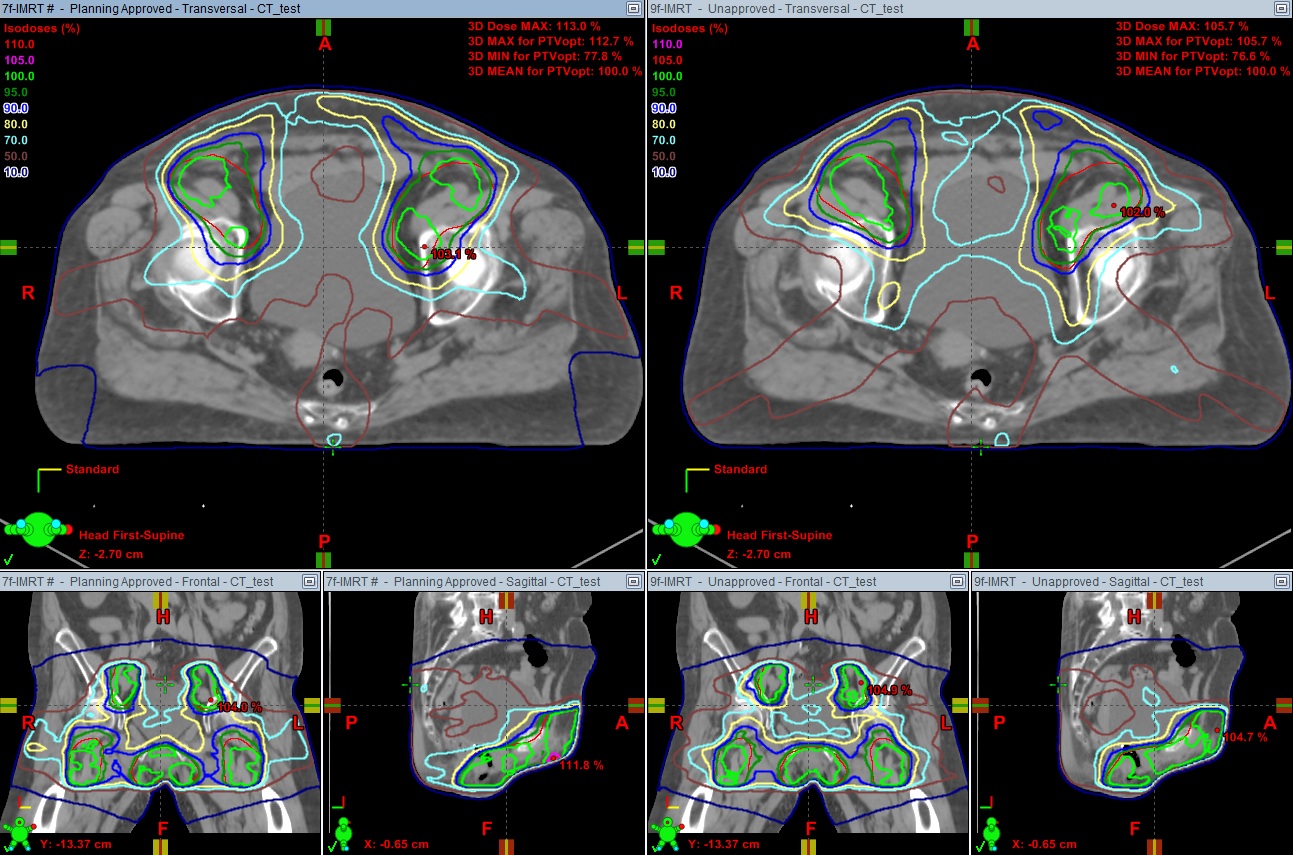

Supplement: Supplementary file 13 — Supplementary Material [file ACM2-17-073-s013.jpg]

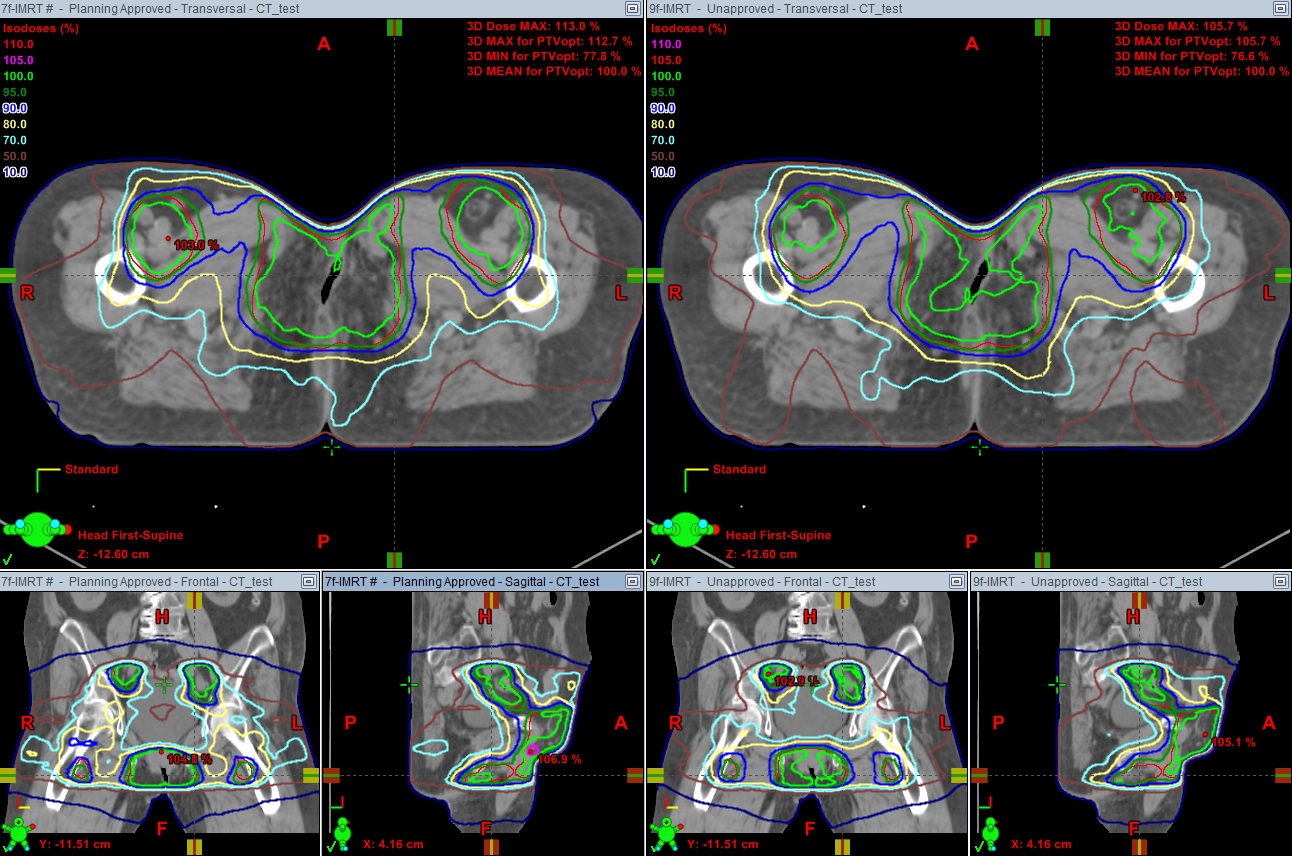

Supplement: Supplementary file 14 — Supplementary Material [file ACM2-17-073-s014.jpg]
